# Somatic mutations in renal cell carcinomas from Chinese patients revealed by targeted gene panel sequencing and their associations with prognosis and PD-L1 expression

**DOI:** 10.1186/s40880-019-0382-8

**Published:** 2019-06-21

**Authors:** Jie Wang, Jianzhong Xi, Hanshuo Zhang, Juan Li, Yuchao Xia, Ruibin Xi, Zhijun Xi

**Affiliations:** 1 Department of Urology, Peking University First Hospital and Institute of Urology, National Research Center for Genitourinary Oncology, No 8, Xishiku Street, Xicheng District, Beijing, 100034 P. R. China; 2 Department of Biomedical Engineering, College of Engineering, Peking University, Beijing, 100871 P. R. China; 3 Beijing Genex Health Technology Co., Ltd, Beijing, 100195 P. R. China; 4 Chongqing Institute of Innovation and Entrepreneurship for Precision Medicine, Chongqing, 401336 P. R. China; 5 School of Mathematical Sciences and Center for Statistical Science, Peking University, 5 Yiheyuan Road, Beijing, 100871 P. R. China

Dear Editor,

Renal cell carcinoma (RCC) is among the most common human cancers in the United States, with approximately 63,990 new patients and 14,400 deaths annually [1]. However, RCC is not among the top 10 malignancies in China in terms of incidence and mortality [2]. The clinical and molecular features of RCC differ among distinct pathological types, mainly clear cell renal cell carcinoma (ccRCC), papillary renal cell carcinoma (PRCC), and chromophobe renal cell carcinoma (ChRCC). The most common subtype of RCC is ccRCC worldwide. According to The Cancer Genome Atlas (TCGA), the somatic mutation landscape of RCC has been revealed by whole-exome sequencing (WES) or whole-genome sequencing (WGS). In our previous WES study, we validated most of the significantly mutated genes reported by the TCGA and identified several novel somatically altered genes [3]. The TCGA study showed that only somatic mutations in BRCA1-associated protein 1 (*BAP1*) were associated with patients’ poor survival outcomes among all significantly mutated genes [4]. In our previous WES study, *BAP1* was somatically mutated in 2 of 15 ccRCC samples [3]. Nevertheless, all of these RCC patients lacked follow-up information. Hence, further analysis is needed to determine whether there are any somatically mutated genes associated with the prognosis of Chinese patients with RCC. However, WES or WGS is time-consuming and costly. Furthermore, compared with targeted sequencing, WES was more likely to generate false positives and false negatives due to insufficient base coverage [5].

In recent years, immunotherapy has played an increasingly important role in the treatment of advanced RCC and other malignancies. Based on the current understanding, programmed death-1 (PD-1) can combine with programmed death-ligand 1 (PD-L1) to confine T cell activity in the tumor microenvironment, and inhibition of the PD-1/PD-L1 pathway can increase the anti-tumor immune response [6]. Nivolumab, a PD-1 immune checkpoint inhibitor, has been validated for the treatment of advanced RCC based on the overall survival (OS) benefit [7]. A recent study has shown that PD-L1 expression was a predictive factor in terms of response and OS benefit from nivolumab plus ipilimumab combination therapy or nivolumab monotherapy as a second-line treatment for advanced RCC [8]. In our previous study, we identified several somatically mutated genes associated with PD-L1 expression in RCC tumor cells, including *CSPG4*, *DNAH11*, *INADL*, and *TMPRSS13* [3]. However, the sample size in the previous study was only 26 specimens, which was a little bit small. In the present study, we aimed to validate these discoveries with a larger sample size and investigate the association between somatic mutations and PD-L1 expression in RCC tumor cells.

In the present study, formalin-fixed paraffin-embedded (FFPE) RCC specimens from 40 patients were investigated using immunohistochemistry (IHC) and targeted sequencing. We designed a gene panel comprising of 173 genes, which contained the newly identified somatically mutated genes, the genes somatically mutated in at least two samples in our previous WES study, and the recurrently mutated genes reported in the TCGA and Catalogue of Somatic Mutations in Cancer (COSMIC) database. The sequencing depth was set to 500×. All the identified somatic mutations were annotated using Annovar [9]. The functional significance of missense mutations was predicted via several algorithms, including SIFT, PolyPhen2 HDIV, PolyPhen2 HVAR, LRT, MutationTaster, MutationAssessor, and FATHMM. The somatic mutations scored with at least two algorithms as deleterious were deemed as deleterious variants. Other variants, including nonsense, frameshift, and canonical ± 1 or ± 2 splice site mutations, were considered to be pathogenic according to the guidelines of the American College of Medical Genetics (ACMG) [10]. Among these 40 RCC patients, 27 were males and 13 were females, with a median age of 57 years (range 22–76 years). The median follow-up for these 40 patients was 74 months (range 15–86 months). Details of their clinicopathological information are listed in Table 1.

**Table 1:** The clinicopathological information of 40 RCC patients

Sample ID	Gender	Age	Subtype	Tumor grade	TNM stage	AJCC stage^a^	OS (months)	DFS (months)	Outcome
1	Male	76	ccRCC	G2	T3aN0M0	III	34	34	Death
2	Male	74	ccRCC	G2	T1aN0M0	I	32	32	Death
3	Male	31	ccRCC	G3	T3aN0M0	III	63	63	Death
4	Male	74	ccRCC	G2	T1aN0M0	I	62	62	Death
5	Male	54	ccRCC	G2	T1aN0M0	I	57	57	Death
6	Male	62	ccRCC	G2	T1aN0M0	I	76	64	Survival (metastasis)
7	Female	40	ccRCC	G3	T3bN0M0	III	74	24	Survival (metastasis)
8	Female	57	ccRCC	G2	T1aN0M0	I	74	24	Survival (metastasis)
9	Male	56	ccRCC	G2	T3aN0M0	III	72	9	Survival (metastasis)
10	Male	59	ccRCC	G3	T1bN0M0	I	71	12	Survival (metastasis)
11	Male	55	ccRCC	G1	T1bN0M0	I	74	74	Survival
12	Male	62	ccRCC	G2	T1bN0M0	I	74	74	Survival
13	Male	54	ccRCC	G2	T1aN0M0	I	74	74	Survival
14	Male	60	ccRCC	G2	T1bN0M0	I	74	74	Survival
15	Male	48	ccRCC	G1	T1aN0M0	I	74	74	Survival
16	Male	68	ccRCC	G2	T1bN0M0	I	73	73	Survival
17	Male	48	ccRCC	G2	T1aN0M0	I	73	73	Survival
18	Male	73	ccRCC	G1	T1aN0M0	I	73	73	Survival
19	Female	58	ccRCC	G2	T1aN0M0	I	72	72	Survival
20	Male	48	ccRCC	G2	T1aN0M0	I	72	72	Survival
21	Male	49	PRCC	G2	T2N0M0	II	15	15	Death
22	Female	66	PRCC	G2	T3aN1M0	III	37	37	Death
23	Male	70	PRCC	G2	T3aN0M0	III	66	66	Death
24	Male	63	PRCC	G2	T3bN0M0	III	29	29	Death
25	Male	65	PRCC	G2	T1aN0M0	I	49	49	Death
26	Female	22	PRCC	G1	T1aN0M0	I	76	76	Survival
27	Female	60	PRCC	G2	T1aN0M0	I	74	74	Survival
28	Male	69	PRCC	G2	T1aN0M0	I	73	73	Survival
29	Male	59	PRCC	G2	T1aN0M0	I	71	71	Survival
30	Male	58	PRCC	G2	T1aN0M0	I	69	69	Survival
31	Female	49	ChRCC	NA	T2N0M0	II	86	86	Survival
32	Male	64	ChRCC	NA	T1aN0M0	I	85	85	Survival
33	Female	37	ChRCC	NA	T1aN0M0	I	85	85	Survival
34	Male	36	ChRCC	NA	T1bN0M0	I	84	84	Survival
35	Female	54	ChRCC	NA	T1aN0M0	I	84	84	Survival
36	Female	52	ChRCC	NA	T1aN0M0	I	82	82	Survival
37	Female	75	ChRCC	NA	T1aN0M0	I	78	78	Survival
38	Male	40	ChRCC	NA	T1bN0M0	I	77	77	Survival
39	Female	36	ChRCC	NA	T1bN0M0	I	77	77	Survival
40	Female	49	ChRCC	NA	T1bN0M0	I	76	76	Survival

*RCC* renal cell carcinoma, *TNM* tumor-node metastasis stage, *AJCC* American Joint Committee on Cancer, *OS* overall survival, *DFS* disease-free survival, *ccRCC* clear cell renal cell carcinoma, *PRCC* papillary renal cell carcinoma, *ChRCC* chromophobe renal cell carcinoma, *NA* not available

^a^The 7th edition of the AJCC Cancer Staging Manual was used

Among all the significantly mutated genes in ccRCC from the TCGA database, *VHL*, *PBRM1*, *SETD2*, *KDM5C*, *PTEN*, *BAP1*, *MTOR*, and *TP53* were the eight most significantly mutated genes [4]. All the eight genes were validated in the present study, whereas only six were validated in our previous WES study [3]. In the present study, *VHL* was somatically mutated in 10 ccRCC specimens, including five frameshift mutations, namely, p. K159fs, p. L135fs, p. P2fs, p. S183fs, and p. R58fs, all of which had not been reported previously and were deemed to be very strong evidence of pathogenicity. *PBRM1* was somatically mutated in 7 ccRCC specimens, 5 PRCC specimens, and 3 ChRCC specimens. Most of the mutations in *PBRM1* were frameshift mutations, which had not been reported previously and were predicted to be deleterious. The tumor mutation burden (TMB) of the 40 RCC specimens was calculated based on the custom-designed 173-gene panel. The TMB was significantly higher in RCC specimens with somatically mutated *PBRM1* than in those without somatically mutated *PBRM1* (*P* = 0.020). The sequencing depth in the present study was higher than that in our previous WES study. Consequently, more somatic mutations in each single specimen were revealed in the present study than in the TCGA data. There was usually more than one type of mutation identified in a single gene in multiple specimens. For instance, *BAP1* was somatically mutated in 3 ccRCC specimens in the present study, namely a frameshift deletion (p. S432fs) and insertion (p. P462fs) in sample 9, a deletion–insertion mutation [p. E642_I643delins (39)] in sample 13, and a frameshift insertion (p. P339fs) and deletion–insertion mutation [p. I191_D192delins (18)] in sample 20. Mutated *BAP1* or loss of *BAP1* expression was reported to be associated with poor outcome in ccRCC [4, 11]. However, no significant association between *BAP1* and prognosis was found in the present study.

In our previous WES study, we identified several newly somatically mutated genes, including *HGC6.3*, *DDX51*, *NWD2*, *CDC42EP1*, *NPIPB5*, *HSCB*, *HMCN2*, and *PCDHB9* in ccRCC; *DEPDC4*, *PNLIP*, *SARDH*, and *ZAN* in PRCC; and *KRTAP4-8* in ChRCC [3]. All of these genes were enrolled in our custom-designed gene panel for further investigation with a larger sample size. As such, most of these newly identified somatically mutated genes were validated in the present study, except for *HMCN2* and *PCDHB9* in ccRCC and *DEPDC4* and *ZAN* in PRCC. Three somatic mutations in *DEPDC4* were identified in ccRCC specimens, namely 2 frameshift deletions (p. F150fs and p. R21fs) and 1 deletion–insertion [p. E147_L148delins (8)], all of which were predicted to be deleterious*.* Among the 40 RCC patients with complete follow-up information, univariate survival analysis with log-rank tests revealed that the disease-free survival (DFS) was shorter in patients with the maximum diameter of tumor > 7 cm than in patients with the maximum diameter of tumor ≤ 7 cm (*P* = 0.003) and shorter in patients with American Joint Committee on Cancer (AJCC) stage III than in patients with AJCC stage I–II (*P* < 0.001). In addition, we found a slight trend towards an association between DFS and somatically mutated *DDX51* (*P* = 0.144). The three variables with *P* < 0.15 were all enrolled in the multivariate Cox regression survival analysis, which showed that somatically mutated *DDX51* (*P* = 0.017) and AJCC stage III (*P* = 0.006) were independent risk factors for DFS among RCC patients (Table 2 and Fig. 1). However, no significant association between somatically mutated genes and OS was found in the present study. Among the 20 ccRCC specimens in the present study, *DDX51* was somatically mutated in 5 specimens with six mutations, namely, a deletion–insertion mutation [p. K611_V612delins (35)] in 1 specimen, a missense mutation (p. S116N) in 2 specimens, two frameshift insertion mutations (p. G147fs and p. H28fs) in 1 specimen, and a frameshift deletion (p.A273fs) in 1 specimen. Notably, the frameshift deletion (p. A273fs) was located in the DEAD protein domain of *DDX51* (Fig. 2). The missense mutation in *DDX51* in both specimens was predicted to be benign or neutral, whereas the deletion–insertion mutation and three frameshift mutations were most likely to be deleterious according to the ACMG guidelines. Furthermore, somatic mutations in *DDX51* were also identified in two other RCC subtypes, including a frameshift deletion (p. R519fs) in PRCC predicted to be deleterious and a missense mutation (p. P123R) in ChRCC predicted to be benign or neutral.

**Table 2:** Multivariate Cox regression analysis for the DFS of RCC patients

Variables	B	Wald	*P* value	Hazard ratio	95% CI
Mutated *DDX51*	1.629	5.696	0.017	5.099	1.338–19.432
AJCC stage III	1.903	7.639	0.006	6.703	1.739–25.833
Maximum diameter of tumor > 7 cm	1.165	2.532	0.112	3.207	0.763–13.475

*DFS* disease-free survival, *RCC* renal cell carcinoma, *CI* confidence interval, *DDX51* DEAD-box helicase 51, *AJCC* American Joint Committee on Cancer

**Fig. 1:**
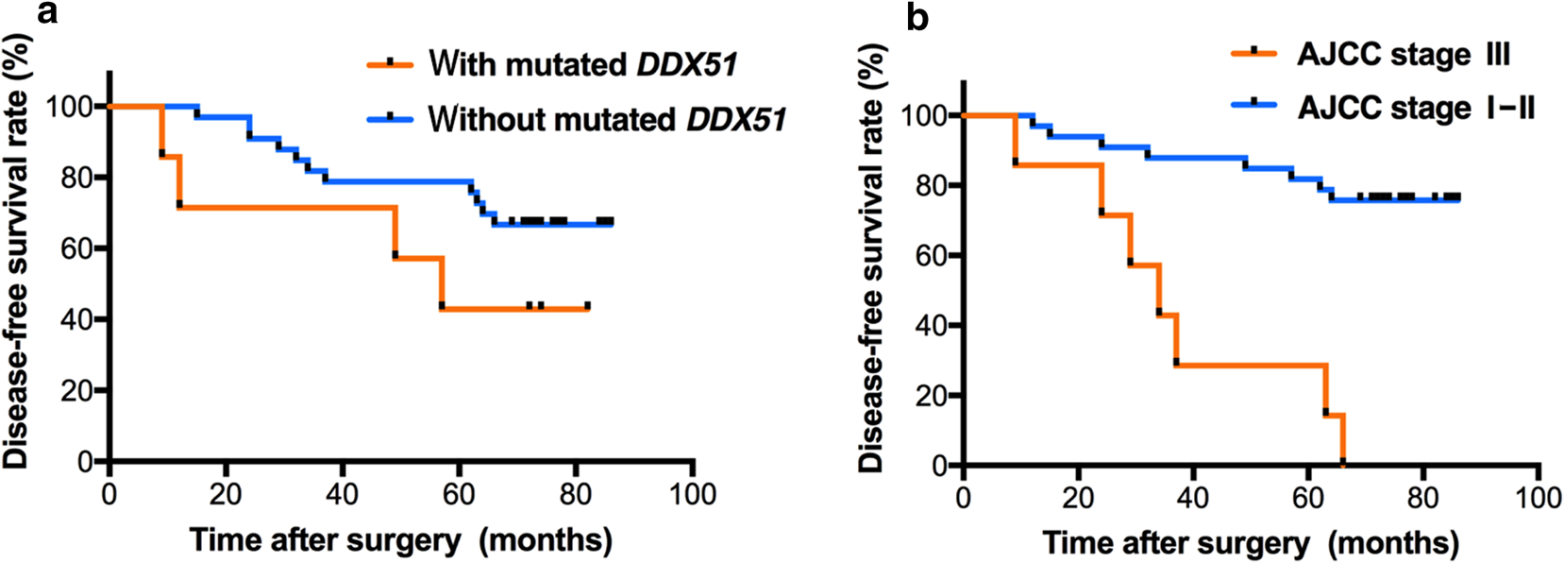
The Kaplan–Meier disease-free survival (DFS) curves of 40 renal cell carcinoma (RCC) patients. **a** Survival curves of patients with or without nutated DEAD-box helicase 51 (*DDX51*); **b** survival curves of patients with American Joint Committee on Cancer (AJCC) stages I–II or III

**Fig. 2:**
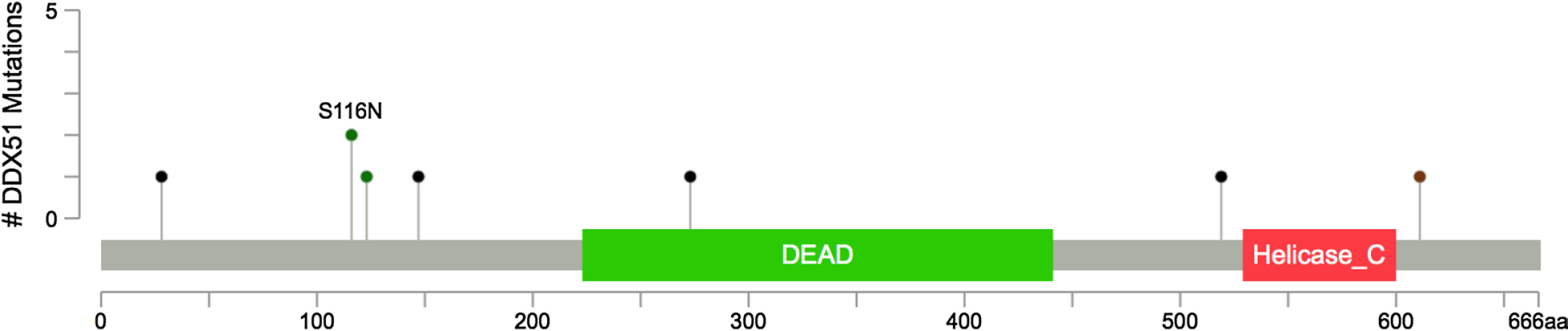
Mutation Mapper interprets mutations with protein domains of DEAD-box helicase 51 (*DDX51*). The mutations are presented by circles and colors: green (missense), black (frameshift), brown (stop-gain)

In our previous study, PD-L1 expression in tumor cells was detected in 6 (23%) of 26 RCC specimens: 3 ccRCC specimens, 2 PRCC specimens, and 1 ChRCC specimen [3]. In the present study, PD-L1 expression in tumor cells was detected in 6 (15%) of the 40 RCC samples: 1 ccRCC sample, 4 PRCC samples, and 1 ChRCC sample (Fig. 3). Combined with the 26 RCC specimens investigated in our previous study, PD-L1 expression in tumor cells was positive in 4 (11%) of 35 ccRCC specimens. We identified 6 genes, *VHL*, *INADL*, *MUC4*, *RAD21*, *CSPG4*, and *BAP1*, that were somatically mutated in 3 of the 4 PD-L1-positive ccRCC specimens. Nevertheless, only mutated *RAD21* and *BAP1* were associated with PD-L1 expression in tumor cells. Among the 35 ccRCC specimens (15 from our previous WES study [3] and 20 in the present study), Fisher's exact test revealed that the PD-L1-positive rate in tumor cells was higher in specimens with somatically mutated *RAD21* (*P* = 0.002) and *BAP1* (*P* = 0.006) than in specimens without those mutated genes. The somatic mutations in *BAP1* (p. P352fs, p. H193Q, p. S432fs, and p. P462fs) and *RAD21* (p. F2 L, p. F304S, p. R402fs, and p. L515fs) detected in the 3 PD-L1-positive ccRCC samples were all predicted to be deleterious.

**Fig. 3:**
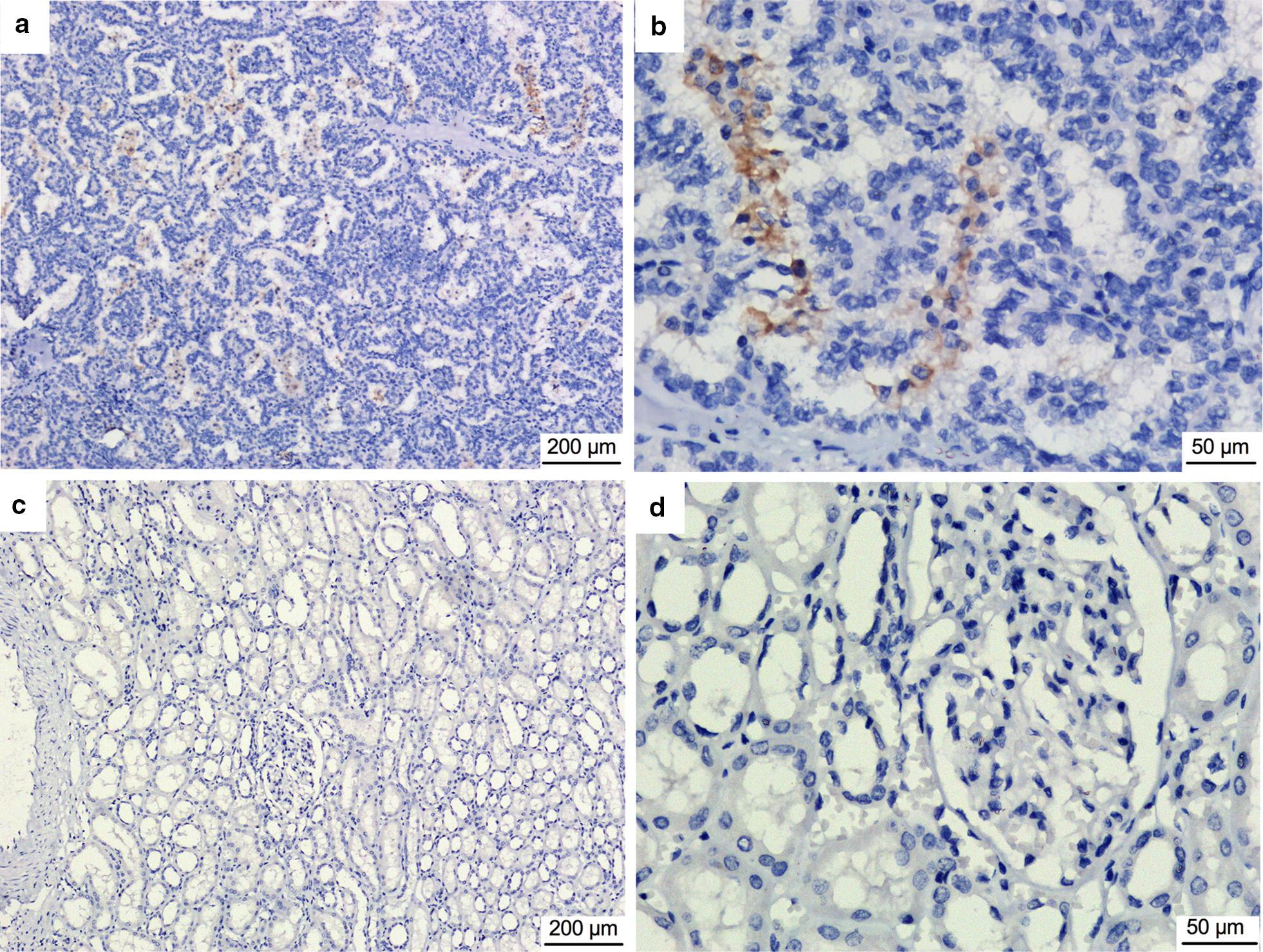
Immunohistochemical staining of programmed death-ligand 1 (PD-L1) in renal cell carcinoma specimens. **a**, **b** Yellowish-brown PD-L1-positive staining on cell membrane in a G1 tumor; **c**, **d** PD-L1-negative staining in adjacent normal tissue

In conclusion, RCC patients with somatically mutated *PBRM1* tend to have higher TMB than those without it. The somatically mutated *DDX51* is an independent risk factor for DFS among RCC patients and could be a new candidate gene for predicting the prognosis of RCC. The somatically mutated *RAD21* and *BAP1* are associated with PD-L1 expression in ccRCC tumor cells and might serve as a potential predictor of the response to immunotherapy with PD-1/PD-L1 inhibitors in ccRCC patients.

## Data Availability

All the raw data generated and analyzed during this study are available from the corresponding author on reasonable request.

## Abbreviations

RCC: renal cell carcinoma
ccRCC: clear cell renal cell carcinoma
PRCC: papillary renal cell carcinoma
ChRCC: chromophobe renal cell carcinoma
TCGA: The Cancer Genome Atlas
WES: whole-exome sequencing
WGS: whole-genome sequencing
PD-1: programmed death-1
PD-L1: programmed death-ligand 1
OS: overall survival
FFPE: formalin-fixed paraffin-embedded
AJCC: American Joint Committee on Cancer
IHC: immunohistochemistry
COSMIC: Catalogue of Somatic Mutations in Cancer
ACMG: American College of Medical Genetics
TMB: tumor mutation burden
DFS: disease-free survival
*BAP1*: BRCA1-associated protein 1
*DDX51*: DEAD-box helicase 51
